# Ameliorative effects of aqueous extract of *Forsythiae suspensa* fruits on oxaliplatin-induced neurotoxicity in vitro and in vivo

**DOI:** 10.1186/s12906-019-2761-8

**Published:** 2019-11-29

**Authors:** Jin-Mu Yi, Sarah Shin, No Soo Kim, Ok-Sun Bang

**Affiliations:** Clinical Medicine Division, Korea Institute of Oriental Medicine, Daejeon, 34054 Republic of Korea

**Keywords:** *Forsythia suspensa*, Forsythoside A, Neuroprotection, Oxaliplatin, Peripheral neuropathy

## Abstract

**Background:**

The dried fruits of *Forsythia suspensa* has generally been used to clear heat and detoxify in traditional Korean and Chinese medicine. Oxaliplatin is a first-line treatment chemotherapeutic agent for advanced colorectal cancer, but it induces peripheral neuropathy as an adverse side effect affecting the treatment regimen and the patient’s quality of life. The present study was conducted to evaluate the neuroprotective effects of an aqueous extract of *F. suspensa* fruits (EFSF) on oxaliplatin-induced peripheral neuropathy.

**Methods:**

The chemical components from EFSF were characterized and quantified using the ultra-high performance liquid chromatography-diode array detector system. The cytotoxicities of anticancer drugs in cancer cells and PC12 cells were assessed by the Ez-Cytox viability assay. To measure the in vitro neurotoxicity, the neurite outgrowth was analyzed in the primary dorsal root ganglion (DRG) cells, and neural PC12 cells that were differentiated with nerve growth factor. To evaluate the in vivo neuroprotective activity, the von Frey test was performed in six-week-old male mice (C57BL/6) receiving EFSF (60–600 mg/kg) in the presence of 20–30 mg/kg cumulative doses of oxaliplatin. Thereafter, the mice were euthanized for immunohistochemical staining analysis with an antibody against PGP9.5.

**Results:**

EFSF attenuated the cytotoxic activities of the various anticancer drugs in neural PC12 cells, but did not affect the anticancer activity of oxaliplatin in human cancer cells. Oxaliplatin remarkably induced neurotoxicities including cytotoxicity and the inhibited neurite outgrowth of DRG and neural PC12 cells. However, the co-treatment of EFSF (100 μg/ml) with oxaliplatin completely reversed the oxaliplatin-induced neurotoxicity. Forsythoside A, the major component of EFSF, also exerted remarkable neuroprotective effects against the oxaliplatin-induced neurotoxicity. In addition, EFSF (60–200 mg/kg) significantly alleviated the oxaliplatin-induced mechanical allodynia and loss of intra-epidermal nerve fiber to the levels of the vehicle control in the mouse peripheral neuropathy model.

**Conclusions:**

EFSF could be considered a useful herbal medicine for the treatment of peripheral neuropathy in cancer patients receiving chemotherapy with oxaliplatin.

## Background

Forsythiae Fructus is listed in the Korean pharmacopoeia as “Yeon-gyo” [1] and in the Chinese pharmacopoeia as “Qingqiao” [2]. It has been prescribed to treat erysipelas, pyrexia, gonorrhea, and ulcers. Many studies have been done on the phytochemicals, pharmacological evaluation and quantitative analyses of *Forsythiae suspensa* [3, 4]. It has also been reported that the crude extracts of *F. suspensa* fruit have protective activities against hepatic injury as well as antibacterial, antiviral, anti-inflammatory, and anti-allergy activities [5–7]. *F. suspensa* contains various phytochemicals including phenylethanoid glycosides, lignans, flavonoids, terpenes, and volatile oils [8–10]. It has been reported that the prominent compounds of *F. suspensa* fruit were identified as forsythoside A (FSA), phillyrin and rutin, and their contents were 2.9, 0.205, and 0.16% on average, respectively [11, 12]. The forsythoside A, one of the phenylethanoid glycosides*,* exerted many protective effects such as the anti-inflammatory, antioxidant, antibacterial, antiviral, and anti-allergic effects [13–16]. Recently, it was demonstrated that the FSA-containing organic fraction of *F. suspensa* fruit exerted neuroprotective effects on rotenone-induced neurotoxicity in PC12 cells as well as in a rotenone-induced neurodegenerative rat model through antioxidant and anti-inflammatory activities, suggesting its application in the treatment of Parkinson’s disease [17]. However, the neuroprotective effect of *F. suspensa* or FSA on chemotherapy-induced peripheral neuropathy (CIPN) is not known yet.

Anticancer drugs such as, taxanes, platinum compounds, vinca alkaloids, and proteasome inhibitors are known to be highly toxic to sensory neurons [18]. CIPN is clinically a common and relevant adverse side effect of anti-cancer agents, and its clinical symptoms are a pain, numbness, prickling, burning, and tingling sensation in the hands and feet [19–21]. Oxaliplatin has been used for the treatment of colorectal cancer. It has been suggested that the metabolites of oxaliplatin, such as the platinum complex may contribute to the development of CIPN [22]. Oxaliplatin-induced peripheral neuropathy (OIPN) is progressive and includes acute or chronic neurotoxicity. A transient and acute OIPN occurs in most cancer patients during or shortly after chemotherapy and is characterized by dysesthesia and paresthesia of the hands, feet, and the perioral region [23]. Chronic OIPN is a dose limiting and cumulative neurotoxicity occurring in 10–15% of cancer patients after continuing exposure to oxaliplatin, and characterized by temperature-insensitive paresthesia, hypoesthesia, and dysesthesia of the hands and feet [24, 25]. Chronic OIPN takes months or years to get over, or even persists throughout life, therefore, it affects a cancer patient’s quality of life and functional status [25, 26].

Because the mechanisms underlying acute and chronic CIPN are still unclear, there are no agents showing efficacy in the prevention or treatment of CIPN. To date, antidepressants, anticonvulsants and chemoprotectants including amifostine, nimodipine and neurotropin are used for symptomatic management of CIPN. However, many treatments are still in the experimental stage and there are not enough clinical evidences to confirm their efficacy [27–31]. Therefore, it is required to establish effective therapeutic strategies for the treatment of CIPN.

In our preliminary screening to develop a novel drug that alleviates neurotoxicity, we found effective materials to relieve OIPN from the library of medicinal herb extracts. In order to assess the neuroprotective effect of the aqueous extracts of *F. suspensa* fruits (EFSF), this study was conducted and showed that the EFSF exerted a neuroprotective potential against OIPN; in both in vitro neuronal differentiated PC12 (neural PC12) cells and in vivo oxaliplatin-induced neuropathic mouse models. Its major component, FSA, also showed a neuroprotective potential against oxaliplatin-induced neurotoxicity in the neural PC12 cells.

## Methods

### Chemicals and reagents

For ultra-high performance liquid chromatography (UHPLC) analysis, analytical-grade formic acid and UHPLC grade solvents were obtained from Fisher Scientific Ltd. (Loughborough, UK). FSA as an authentic standard chemical (STD) was purchased from ChemFaces (Wuhan, Hubei, China), and its chemical purity was > 98% according to the manufacturer’s information sheet. Oxaliplatin, bortezomib and amifostine were purchased from TOCRIS Bioscience (Bristol, UK), Cell Signaling Technology (Danvers, MA, USA) and Santa Cruz Biotechnology (Dallas, TX, USA), respectively. Cisplatin, paclitaxel, docetaxel, and vincristine were purchased from Sigma-Aldrich Co (St. Louis, MO, USA). Recombinant rat nerve growth factor (NGF) and N2 supplement were purchased from R&D systems (Minneapolis, MN, USA) and Thermo Fisher Scientific (Waltham, MA, USA), respectively. A rabbit antibody against PGP9.5 and a goat anti-rabbit IgG Alexa Flour 488-labeled antibody were purchased from Millipore (Temecula, CA, USA) and Abcam (Brandford, CT, USA), respectively.

### Preparations of herbal extract

The dried fruits of *F. suspensa* were supplied from Kwangmyung-dang Medicinal Herbs Co. (Ulsan, Republic of Korea), and their morphology was carefully validated by Dr. Goya Choi Herbal Research Specialized Center, Korea Institute of Oriental Medicine (KIOM), Republic of Korea. A voucher specimen (KIOM010016) was deposited in the Clinical Medicine Division of KIOM. The herb materials were extracted as previously described [32]. Briefly, the dried materials (1 kg) were refluxed in distilled water (10 L) for 3 h, twice. The extracts were filtered, concentrated using a rotary evaporator (N-1200A, Tokyo, Japan), freeze dried using a freeze drier (FD8518, IlshinBioBase, Dongduchun, Republic of Korea), and then, homogenized. The final product, EFSF was stored under desiccated condition until use.

### Chromatographic analysis

For a quality control of EFSF, the components of EFSF were analyzed using an UHPLC-diode array detector (DAD) system (1290 infinity, Agilent Technologies, CA, USA) equipped with an analytical column (Luna Omega C18, 2.1 × 50 mm, 1.6 μm, Phenomenex, CA, USA) maintained at 30 °C. The EFSF (2 mg/ml) and STD of FSA (0.1 mg/ml) were dissolved in 50% (v/v) methanol in water. The EFSF or STD was separated by using a sequential gradient mobile phase system from 90% of 0.1% formic acid (A) and 10% of acetonitrile (B) to 65% A and 35% B within 40 min at a flow rate of 0.17 ml/min. The eluent signals were monitored with a DAD at 330 nm. Each component from EFSF was verified by comparing the retention time (t_R_) and specific DAD spectrum pattern of each peak in the EFSF with those of FSA in parallel. The acquired chromatographic data were analyzed by the Agilent OpenLAB CDS software.

### Cell culture and viability assay

PC12 (CRL-1721), A549 (CCL-185), and HCT-116 cells (CCL-247) were purchased from the American Type Culture Collection (Rockville, MD, USA). Rat dorsal root ganglion neurons (RDRGN, Cat**#** R8820NK) were purchased from Cell Applications, Inc. (San Diego, CA, USA). PC12, rat adrenal gland pheochromocytoma cells were cultured in a collagen type I-coated culture dish (Corning, Bedford, MA, USA) with DMEM supplemented with 10% horse serum and 5% fetal bovine serum (FBS). According to the manufacture’s instruction, DRG cells were cultured at a density of 2 × 10^3^ cells/well in the Poly-D-Lysine (PDL)-coated 96 well plate with Rat Ganglion Neuron Culture Medium. For neuronal differentiation of the PC12, the cells were plated in a collagen type IV-coated multi-plate (Corning) at the density of 2.5 × 10^3^ cells for a 96 well plate or 1.25 × 10^4^ cells for a 24 well plate. After 24 h, the culture medium was replaced with differentiation medium (DM) consisting DMEM, 100 ng/ml NGF, 1% N2 supplement, and 0.5% FBS, and incubated for 4 days.

For the cell viability assay, neural PC12 cells were exposed to the indicated concentrations of EFSF, FSA, each anticancer drug, or a combination of each anticancer drug with EFSF or FSA for 48 h. Amifostine was used as a positive control. Cell viability was assessed using the Ez-Cytox viability assay kit (Daeil Lab Service Co., Seoul, Republic of Korea). Human cancer cells, A549 (lung cancer) and HCT-116 (colon cancer) cells were cultured in 96-well plates (5 × 10^3^ cells/well) with RPMI medium supplemented with 10% FBS. The effect of EFSF on the values of the half maximal inhibitory concentration (IC_50_) of oxaliplatin was assessed in cancer cells co-treated with oxaliplatin (0–250 μM) and EFSF (0–100 μg/ml) for 48 h, and the IC_50_ values were determined using the SoftMax Pro 7.0 software (Molecular Devices, Sunnyvale, CA, USA) automatically.

### Neurite outgrowth assays

Neurite outgrowth in PC12 and DRG cells were analyzed as described previously [33]. To investigate the effect of EFSF and FSA on the oxaliplatin-induced neurotoxicity, PC12 cells (1.25 × 10^4^ cells/well) were cultured in DM with a combination of oxaliplatin (200 nM) and EFSF (0–100 μg/ml), FSA (0–40 μM) or amifostine (0.5 mM). The neurite outgrowth was observed under a phase contrast bright-field inverted microscope (IX71, Olympus, Tokyo, Japan). Digitalized morphometric images of the fields containing more than 20 cells were captured and used for the determination of the length and number of neurites per cells. The cells bearing neurite that had at least one neurite with a length longer than the diameter of the cell body were calculated as a percentage of the total of counted cells. Total neurite lengths were measured by tracing the length of the neurites using the MetaMorph image software (Molecular Devices).

The neurite outgrowth assay in DRG cells was conducted using the Neurite Outgrowth Assay Kit (NS225, Millipore, Billerica, MA, USA). Briefly, the DRG cells (4 × 10^4^ cells) were cultured with growth factor-containing media in the PDL pre-coated Millicell inserts, and treated with the indicated concentrations of EFSF in the presence or absence of oxaliplatin (5 μM). After 7 days of the culture, the cells were fixed with − 20 °C methanol for 20 min at room temperature and then, the neurites were stained with the Neurite Stain Solution for 30 min. The extended neurites on the underside of the insert membrane were visualized using inverted microscope. To extract the stained neurites, cell bodies were removed with cotton swab and then the stain was extracted on Parafilm using Extraction Buffer for 10 min at room temperature. Each extract was transferred to 96 well plate and quantified neurite extension on a multi-plate reader at 562 nm.

### Experimental animals and drug administration

Six-week-old male mice (C57BL/6) were supplied by OrientBio, Inc. (Seongnam, Republic of Korea). During the entire experimental periods, the mice were housed under a specific-pathogen-free laboratory animal care facility (an alternating 12 h day-night cycle, at 22 ± 2 °C and 45 ± 10% relative humidity), and received food and water ad libitum*.* They were acclimated for 1 week before the treatments. All procedures for animal care and experiments were in accordance with the relevant national raw and approved by the Institutional Animal Care and Use Committee (Protocol #16–036, 16–089) of KIOM.

To generate the chronic OIPN model, the mice received an intraperitoneal (i.p.) injection of 5% dextrose solution (vehicle) or oxaliplatin (5 mg/kg in 5% dextrose) twice a week, for 3 weeks (total 30 mg/kg). After 3 weeks from the first injection, the vehicle group received a peroral (p.o.) administration of 0.5% Na-carboxymethyl cellulose (CMC) solution throughout the experimental period. To determine the optimal effective dose of EFSF in the behavior test, oxaliplatin-administered mice were randomly divided into 4 groups. Each group (*n* = 6) daily received oral administration of 0.5% Na-CMC (OIPN model group), or three doses of 60, 200 or 600 mg/kg EFSF (treatment group) throughout the experimental period, respectively. The neuroprotective effect of EFSF was confirmed by measuring the response rate to mechanical stimuli in another neuropathic animal model. Oxaliplatin (10 mg/kg in 5% dextrose) was i.p. injected into experimental mice once a week, for 2 weeks (total 20 mg/kg), and then the animals were divided into 2 groups. Each group (*n* = 6) received a daily oral administration of EFSF (250 mg/kg) or 0.5% CMC solution for 3 weeks. Forsythiae Fructus has been used at 3–15 g that was a daily dosage for anti-inflammatory and detoxifying treatment [34]. This dosage in a human with an average body weight of 60 kg was converted to 13–65 mg/kg of EFSF (26.1% yield). Therefore, in this mouse experiment, we picked an EFSF dose range from 60 to 600 mg/kg as a starting dose for the OIPN model [35].

### Assessment of mechanical allodynia

The pain sensitivity as a behavioral outcome was evaluated by measuring the response rate to external mechanical stimuli using the plantar von Frey instrument or an electronic von Frey tester. All tests were operated without any information about the drug treatment in the controlled behavior test room. Each animal was placed in a cage with a mesh-like floor and acclimated for 15–30 min before testing.

To evaluate mechanical allodynia to external mechanical stimuli, the stimulation force was measured using the Dynamic Plantar Aesthesiometer (DPA, Ugo Basile, VA, Italy) equipped with a von Frey-type 0.5 mm diameter filament. The mechanical stimuli were applied perpendicularly on to the mid-plantar of the hind paw with a gradual increase in the force (app. 1 g/sec, cut-off force 10 g) until the mouse withdrew its hind paw. The test was performed on both sides of the hind paw and repeated 5 times with an interval of 5 min between each test cycle. The withdrawal threshold force was defined as the force (g), at which the mouse withdrew its paw.

To measure the responses rates of the mouse to the external stimuli, a von Frey monofilament with a bending force of 0.16 or 0.4 (DanMic Global, San Jose, CA, USA) was applied to the mid-plantar surface of the hind paw, 6 times for 3 s with an interval of 10 s between stimulations. The test was performed in 3 cycles with an interval of 15–20 min between each test cycle. The responses such as rapid and sudden lifting, shaking or licking were counted as a positive response. The response rate was calculated as the percentage of positive responses from a total of 6 trials in each cycle. After the final test, the animals were anesthetized by i.p. injection of a pentobarbital solution (50 mg/kg) followed by transcardinal perfusion of 4% paraformaldehyde (PFA).

### Immunohistochemical staining

The hind footpads were removed from the mice and fixed in 4% PFA. The PFA-fixed tissues were embedded in paraffin, sectioned at a 4 μm thickness, and mounted on silane-coated slides (Muto Pure Chemicals, Tokyo, Japan). After deparaffinization, intra-epidermal nerve fiber (IENF) was immunostained with a primary rabbit antibody against PGP9.5 (1: 200) at 4 °C overnight. After incubation with the secondary antibody labeled with Alexa Flour 488 for 1 h at room temperature, the slides were covered with a mounting medium (Vectashield, Vector laboratories, Burlingame, CA, USA). The photographed images under a fluorescence microscope (BX41, Olympus, 400X magnification) were analyzed with image software (Cellsense, Olympus). To quantify the nociceptive IENF density, the numbers of nerve fibers that cross the dermal/epidermal junction were counted from 3 randomly chosen fields for each slide and the length of the epidermis within each field was measured. The density was calculated as the value of the IENF numbers divided by the length (mm) in the intra-epidermis.

### Statistical analysis

Statistically significant differences in the averages among the experimental groups were determined by conducting one- or two-way analysis of variance (ANOVA) according to the experimental design. Tukey’s post hoc multiple comparisons test was done to compare the significant differences between the treatment and vehicle groups. All statistical analyses were performed using the SigmaPlot 13.0 software (Systat Software, San Jose, CA, USA).

## Results

### Chromatographic analysis of EFSF

**Fig. 1:**
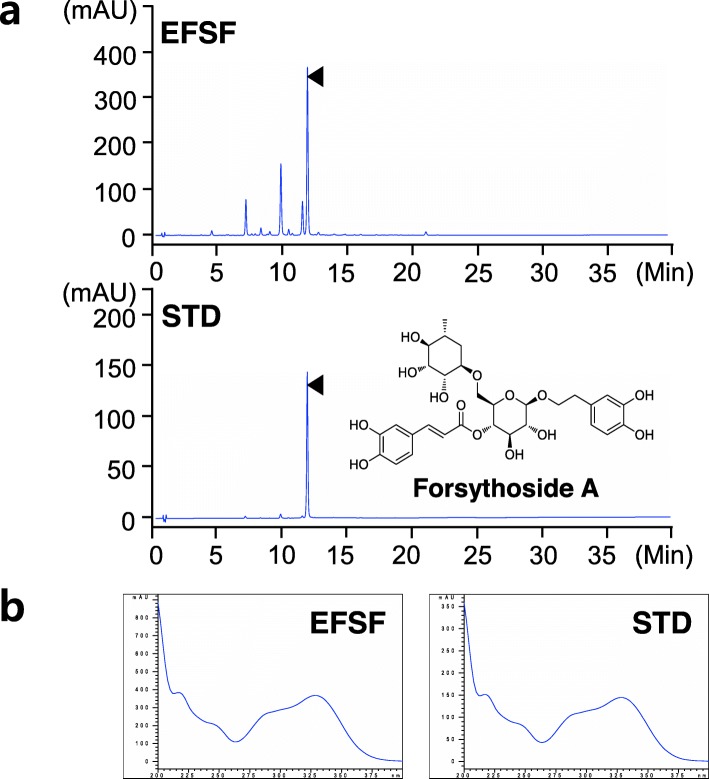
**a** UHPLC-DAD chromatograms of EFSF (upper panel) and its major component, Forsythoside A (lower panel). The structure of FSA is shown in the lower panel. **b** The DAD scanning spectrum of major peak on EFSF and a peak on FSA at 200–400 nm

EFSF was prepared by water extraction of *F. suspensa* fruits with 26.1% yield, and its drug extract ratio was 4:1. The components of the EFSF were analyzed using the UHPLC-DAD. To determine the major components of the EFSF, its UHPLC chromatogram was scanned and compared to the STD peak of FSA which has been described as a marker component of Forsythiae Fructus in the Korea and Chinese Pharmacopoeia [1, 2]. As shown in Fig. 1, the major peak of EFSF (t_R_ = 11.85, upper) and FSA (t_R_ = 11.86, lower) was perfectly matched in the chromatograms. Its content was determined as 124.98 ± 0.13 mg/g based on the calibration curve from 0.5 μg/ml to 0.2 mg/ml of the peak area in the STD chromatogram (Fig. 1a). In addition, the specific DAD spectrum of the major peak in EFSF also showed perfectly the same pattern as that of FSA (Fig. 1b). The results indicate that EFSF contains about 12.5% of FSA, and that FSA can be used as a marker component of EFSF.

### Effect of EFSF on the anticancer drug-induced cytotoxicity

**Fig. 2:**
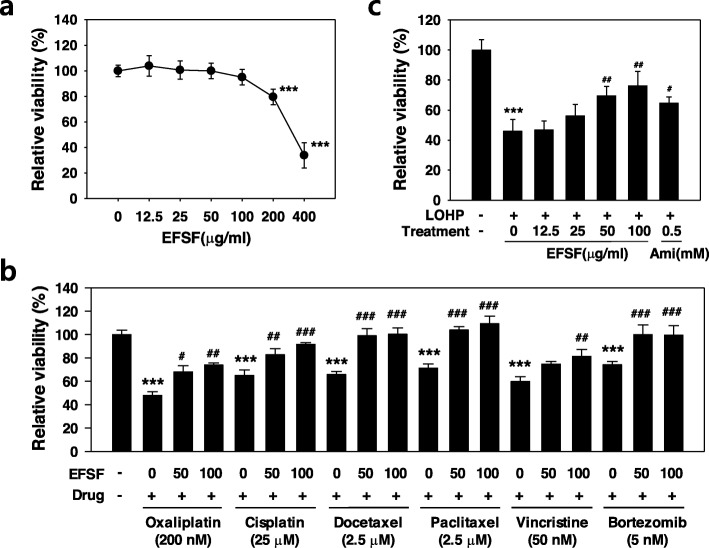
EFSF could relieve the anticancer drug-induced neurotoxicity. PC12 cells were differentiated into neural cells in DM for 96 h as described in the Methods, and treated with various concentrations of EFSF (0–400 μg/ml) (**a**) or with a combination of the indicated anticancer drugs and EFSF (**b**) for 48 h. **c** The neural PC12 cells were co-treated with the oxaliplatin (LOHP, 200 nM) and indicated concentrations of EFSF (0–100 μg/ml) or amifostine (Ami, 0.5 mM) as a positive control for 48 h. Cell viability was assessed after 48 h drug treatment. The data from triplicate experiments are presented as the mean ± SD. *** p < 0.001 vs. vehicle, and ^*#*^ *p* < 0.05, ^*##*^ *p* < 0.01, ^*###*^ *p* < 0.001 vs. anticancer drug (**b**) or LOHP alone (**c**)

To select the dose ranges of EFSF to treat the neural cells, PC12 cells were cultured in DM for 96 h and then cultured with various concentrations of EFSF (0–400 μg/ml) for a further 48 h. EFSF did not significantly change the cell viability of the neural PC12 cells at a range from 0 to 100 μg/ml, but significantly decreased it at concentrations above 200 μg/ml EFSF (Fig. 2a). Therefore, we used EFSF at dose ranges between 0 and 100 μg/ml for further in vitro experiments. To examine the effect of EFSF on the cytotoxicities of anticancer drugs, neural PC12 cells were co-treated with EFSF (0–100 μg/ml) and anticancer drugs, such as oxaliplatin (200 nM), cisplatin (25 μM), paclitaxel (2.5 μM), docetaxel (2.5 μM), vincristine (50 nM), or bortezomib (5 nM) for 48 h, and then the cell viability of neural PC12 cells was measured. As shown in Fig. 2b, all the treated anticancer drugs induced significant cytotoxicities in the neural PC12 cells, which were attenuated by the co-treatment of EFSF (50 and 100 μg/ml). We next further assessed the protective effect of EFSF on the oxaliplatin-induced neurotoxicity. The neural PC12 cells were co-treated with oxaliplatin (200 nM) and various concentrations of EFSF (0, 12.5, 25, 50 and 100 μg/ml) for 48 h. Amifostine (0.5 mM), known as a cytoprotective agent for CIPN [36], was used as positive control. Oxaliplatin dramatically reduced the cell viability of the neural PC12 cells to 46.0 ± 7.7% compared to the vehicle. Co-treated EFSF attenuated the oxaliplatin-induced cytotoxicity in the neural PC12 cells in a dose-dependent manner (Fig. 2c). As expected, amifostine also attenuated the oxaliplatin-induced neuronal cytotoxicity (Fig. 2c). To investigate the effect of EFSF on the antitumor activity of oxaliplatin, human colorectal (HCT-116) and lung (A549) cancer cells were co-treated with various concentrations oxaliplatin (0–250 μM) and EFSF (0–100 μg/ml) for 48 h, and the cell viability was determined. As shown in Table 1, EFSF did not significantly change the IC_50_ values of oxaliplatin indicating that EFSF could alleviate the oxaliplatin-induced neurotoxicity without affecting its antitumor activity.

**Table 1:** Effect of EFSF on the cytotoxic profile of oxaliplatin in human cancer cell lines

Treatment		IC_50_ (μM)^1^	
		A549	HCT-116
Oxaliplatin	Vehicle	4.39 ± 0.27	0.87 ± 0.06
	EFSF (10 μg/ml)	4.13 ± 0.26	0.84 ± 0.09
	EFSF (30 μg/ml)	4.34 ± 0.33	0.87 ± 0.08
	EFSF (100 μg/ml)	4.41 ± 0.23	0.89 ± 0.08

^1^ IC_50_ values are presented as the mean ± SD from the triplicate experiments

### Effect of EFSF on oxaliplatin-induced neurotoxicity in neural PC12 cells

**Fig. 3:**
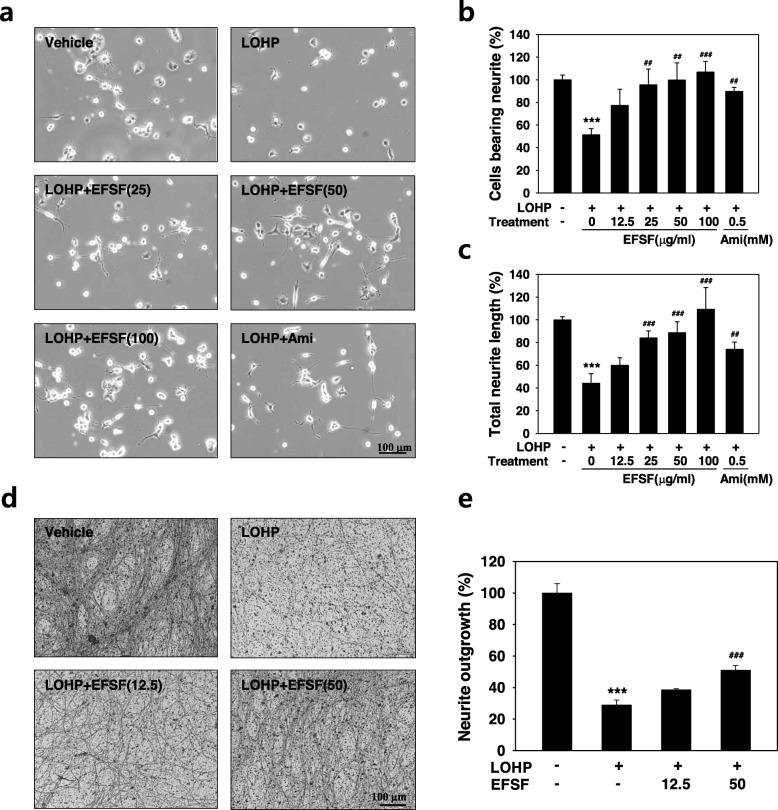
EFSF exerted protective effect against the oxaliplatin-induced neurotoxicity. (**a-c**) The PC12 cells were differentiated in the DM as described in the Methods, and co-treated with oxaliplatin (LOHP, 200 nM) and the indicated concentration of EFSF or amifostine (0.5 mM) for 72 h. (**a**) On day 3 of the during treatment, images were photographed under an inverted microscope (× 200 magnification) and the neurites sprouting were counted. The number of neural PC12 cells extending neurites (**b**) and their total neurite lengths (**c**) were analyzed using an image software. The data from at least triplicate experiments represent the mean ± SD. *** p < 0.001 vs. vehicle. ^*##*^ *p* < 0.01, ^*###*^ *p* < 0.001 vs. LOHP alone. (**d**&**e**) Rat DRG cells (4 × 10^4^) were cultured on the insert with 1 μm pores membrane and treated with LOHP (5 μM) and/or the indicated concentration of EFSF for 7 days. **(d**) The neurites of DRG cells were stained with Neurite Stain Solution, and the representative images were photographed under an inverted microscope (× 200 magnification). (**e**) The extracts of stained neurites were quantitated using a multi-plate reader at 562 nm, and neurite outgrowth was presented as a relative percentage compared to vehicle. ** *p* < 0.01 vs. vehicle, *** *p* < 0.001 vs. vehicle, ^###^ *p* < 0.001 vs. LOHP alone

To investigate the effect of oxaliplatin and EFSF on neurite outgrowth, PC12 cells were treated with oxaliplatin and the EFSF (0–100 μg/ml) or amifostine (0.5 mM), and cultured for 72 h in DM. The PC12 cells were differentiated to neural cells which exhibited extensive neurites sprouting out from the cell bodies as seen in the untreated vehicle of Fig. 3a. Oxaliplatin induced the retraction of the neurite outgrowth from the cell bodies, indicating the neurotoxicity. The numbers of neural PC12 cells extending neurites that were longer than the cell body were counted and then compared to those of the untreated vehicle. Total length of neurites from four captured images of untreated vehicle was measured as 1452 ± 40.0 μm/mm^2^. The relative neurite outgrowth was calculated as a percentage compared to the total neurite length of vehicle. Oxaliplatin induced a strong neurotoxicity showing remarkable decreases in both the number of neurite-bearing cells (51.5 ± 5.4%) and the total lengths of the neuritis (44.3 ± 8.3%) compared with the untreated vehicle. EFSF significantly attenuated the oxaliplatin-induced neurotoxicity, which was dose-dependent. Moreover, EFSF at dose of 100 μg/ml completely alleviated the oxaliplatin-induced neurotoxicity based on both the number of cells bearing neurites and the total neurite lengths (Fig. 3b and c). To investigate whether EFSF has neuroprotective effects on the oxaliplatin-induced neurotoxicity in primary neuron cells, we next performed the neurite outgrowth assay using DRG cells isolated from spinal cords of normal embryonic rat. Primary DRG cells sprouted out much longer neurite than that of differentiated PC12 cells and formed complex neurite network after 7 days of the culture as shown in Fig. 3d. Oxaliplatin dramatically decreased the neurite outgrowth of the DRG cells to 28.9 ± 3.2% compared with untreated vehicle. EFSF significantly restored the oxaliplatin-induced neurotoxicity in a dose-dependent manner in the DRG cells (Fig. 3e). The data confirm that EFSF alleviates oxaliplatin-induced neurotoxicity in primary DRG neurons.

### Effect of the FSA on the oxaliplatin-induced neurotoxicity

**Fig. 4:**
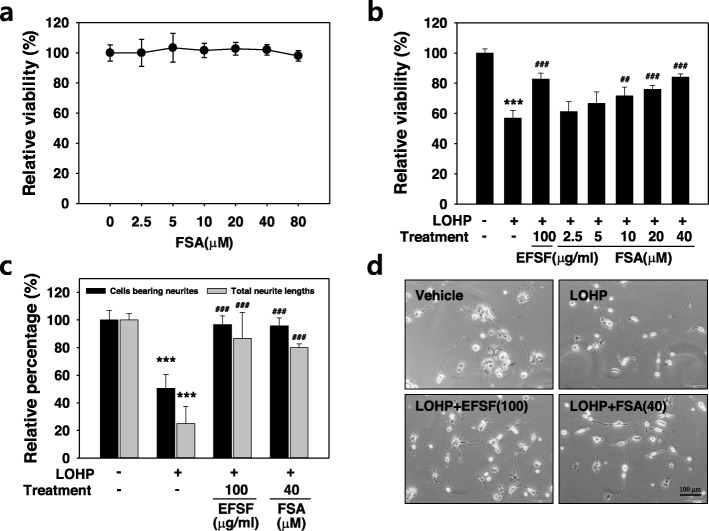
FSA exerted protective effect against the oxaliplatin-induced neurotoxicity. (**a**&**b**) The PC12 cells were differentiated in the DM for 96 h, and then treated with the various concentrations of FSA (0–80 μΜ) (**a**) or a combination of oxaliplatin (LOHP) and the indicated concentrations of FSA or EFSF (**b**). After treatment for 48 h, cell viability was assessed. (**c**&**d**) The neural PC12 cells were treated with the EFSF (100 μg/ml) or FSA (40 μΜ) in the presence of LOHP in the DM. The percentages of neural PC12 cells extending neurites (black bar) and their total neurite lengths (grey bar) were calculated using an image software. The data from at least triplicate experiments are presented as the mean ± SD. Images were photographed under an inverted microscope (× 200 magnification). *** p < 0.001 vs. vehicle control, ^*##*^ *p* < 0.01, ^*###*^ *p* < 0.001 vs. LOHP alone

To investigate the effect of the FSA, the major component of EFSF on the viability of neural cells, the PC12 cells were differentiated in DM for 96 h and then, treated with the indicated concentrations of FSA (0–80 μM) for 48 h. FSA alone did not change the viability of the neural PC12 cells at all the tested concentrations (Fig. 4a). Next, to assess the effect of the FSA on the oxaliplatin-induced neurotoxicity, the neural PC12 cells were treated with oxaliplatin in the absence or presence of FSA (2.5–40 μM) which corresponds to the dose contained in 12.5–200 μg/ml EFSF. FSA significantly increased the cell viability in a dose-dependent manner. Interestingly, FSA at 40 μM dramatically attenuated the oxaliplatin-induced neurotoxicity to a similar level of the 100 μg/ml EFSF treatment (Fig. 4b). When the effect of the FSA on neurite outgrowth was examined after co-treatment with oxaliplatin and FSA (40 μM), FSA remarkably alleviated the oxaliplatin-induced neurotoxicity based on both the number of cells bearing neurites and the total length of neurites, which was similar to the level of EFSF at 100 μg/ml (Fig. 4 c and d). The results indicate that the FSA is a major effective component of EFSF in terms of neuroprotection.

### Effect of EFSF on the oxaliplatin-induced mechanical hypersensitivity

**Fig. 5:**
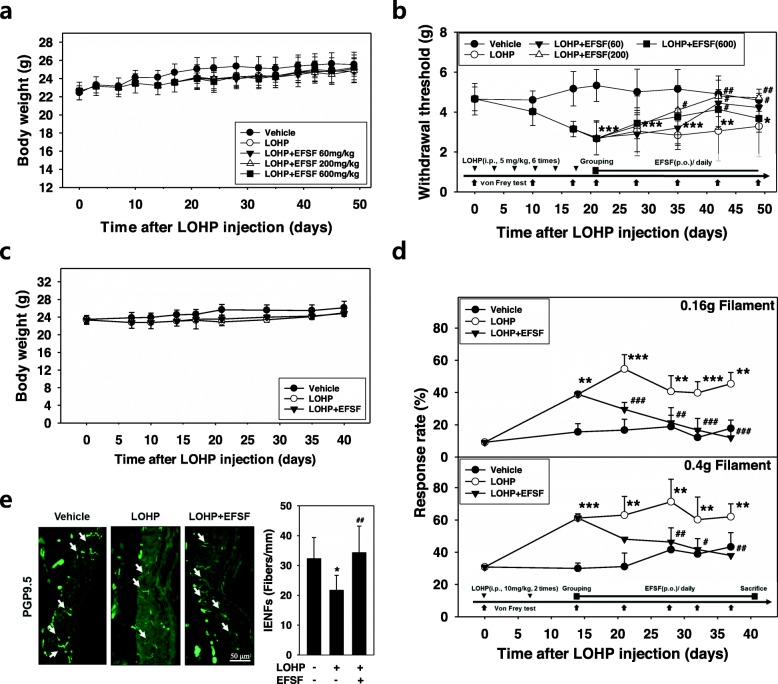
EFSF protected from oxaliplatin-induced the mechanical hypersensitivity and neuronal damage in peripheral skin tissues. **(a**&**b**) Mice repeatedly received a low dose oxaliplatin (LOHP, 5 mg/kg, i.p., 2 times a week, 3 weeks) or 5% dextrose solution as depicted experimental schedule. Three weeks after first LOHP injection, EFSF (60, 200, and 600 mg/kg, p.o.) or 0.5% Na-CMC solution (vehicle) were administered to each group (*n* = 6) for 3 weeks. (**a**&**c**) Body weights were measured every 3 or 4 days throughout the experiment. (**b**) Withdrawal threshold (g) was assessed by the aesthesiometer as described in the Methods. Each point represents the mean ± SD of 6 mice. * p < 0.05, ** p < 0.01, *** p < 0.001 vs vehicle group and ^*#*^ *p* < 0.05, ^*##*^ *p* < 0.01 vs LOHP group. (**c-e**) Mice in each group (n = 6) repeatedly received a low dose of oxaliplatin (LOHP, 10 mg/kg, i.p., once a week, 2 weeks) and EFSF (250 mg/kg, p.o.) or vehicle (5% dextrose, p.o.) as depicted in the experimental schedule. (**d**) At the indicated time point, mechanical stimuli were applied on the mid plantar surface of both paws of the animals using a 0.16 or 0.4 g von Frey monofilament. The responses rates of the hind paws to external mechanical stimuli were calculated and presented as the mean ± SD of six mice. ** p < 0.01, *** p < 0.001 vs. vehicle group and ^*#*^ *p* < 0.05, ^*##*^ *p* < 0.01, ^*###*^ *p* < 0.001 vs LOHP group. (**e**) After final behavior test, the hind paw footpads of the mice were sectioned and subjected to immunohistochemical analysis using an antibody against PGP9.5. IENFs are indicated by arrows (left panel, × 400 magnification). The numbers of IENFs and lengths of epidermis were determined under a fluorescence microscopy. The number of IENFs within 1 mm epidermis, that is IENF densities, were calculated (right panel, fibers/mm). * p < 0.05 vs. vehicle group. ^*##*^ *p* < 0.01 vs. LOHP alone

To determine the effective dose of EFSF for the neuroprotective effect in OIPN, mice were administered 5 mg/kg, 6 times over 3 weeks (accumulative dose, 30 mg/kg). The mice exhibiting mechanical hypersensitivity in the von Frey test were divided into 4 groups. Each group (*n* = 6) received daily EFSF (60, 200 or 600 mg/kg) or 0.5% (w/v) Na-CMC solution. As shown in Fig. 5a, any clinical signs related to toxicity such as sudden death or remarkable body weight loss was not observed in all animal groups during the whole experimental period. Next, to examine the effect of EFSF on the oxaliplatin-induced mechanical hypersensitivity, mechanical allodynia was evaluated by measuring the nociceptive withdrawal threshold using the electronic von Frey tester. The withdrawal threshold of the mice was gradually decreased in response to the oxaliplatin treatment, from 4.9 ± 0.3 g to 2.7 ± 0.8 g by week 3 after the first oxaliplatin injection and then persisted over 4 weeks. The withdrawal threshold of the vehicle group was consistently maintained at the basal level, 4.9 ± 0.3 g during the whole experiment period. When the mice were administered with the indicated doses of EFSF for 3 weeks after the final oxaliplatin injection, the EFSF increased the withdrawal threshold: 60 and 200 mg/kg of EFSF dose-dependently increased the withdrawal threshold up to the vehicle level. However, the 600 mg/kg of EFSF treatment was gradually increased the withdrawal threshold, and then decreased it to the level of the oxaliplatin treatment (Fig. 5b). The results indicated that the effective dose of EFSF for protection against the neurotoxicity of oxaliplatin was between 60 and 200 mg/kg.

To confirm the neuroprotective effect of EFSF against OIPN, the mechanical hypersensitivity was determined by measuring the response rate to external mechanical stimuli using the von Frey monofilaments in the neuropathic mice induced by oxaliplatin injection (accumulative dose, 20 mg/kg). There was no body weight change in the EFSF treatment group compared with the oxaliplatin-only treatment group during the experiment (Fig. 5c). The response rates to mechanical stimuli by von Frey filaments in each treatment group was calculated by counting the number of times showing the withdrawal of left or right hind. At 2 weeks after the treatment of oxaliplatin, the response rates to mechanical stimuli by 0.16 or 0.4 g bending forces were dramatically increased up to 38.9 ± 1.7 and 61.2 ± 2.6%, respectively, and after then, remained at similar levels to the end of the experiment. Therefore, the results indicate that oxaliplatin induced peripheral neuropathy showing increased sensitivity in response to the mechanical stimuli. Co-treatment of EFSF (250 mg/kg) gradually reduced the mechanical hypersensitivity induced by oxaliplatin, and then finally returned it to the basal level as observed in the vehicle group (Fig. 5d).

We next investigated whether oxaliplatin can reduce the density of innervated nociceptive nerve fibers in the distal foot skin, which is a common lesion related to chemotherapy-induced neuropathies [37]. To assess the innervation of the peripheral nerves in the distal foot skin, nociceptive IENFs was immunostained with an antibody against cytoplasmic a pan-axonal marker, PGP9.5. For quantitative analysis of the density, the IENF was counted under the fluorescence microscope (Fig. 5e). In the vehicle group, the IENFs showed a regular penetration through the epidermal-dermal junctions, and their density was 32.3 ± 7.1 fibers/mm. The oxaliplatin treatment induced a remarkable loss in the IENF density by 21.7 ± 5.0 fibers/mm. However, EFSF treatment recovered the oxaliplatin-induced loss of IENF to a density of 34.3 ± 8.9 fibers/mm which was comparable to that of vehicle treatment group.

## Discussion

In our preliminary screening to find a novel drug for the treatment of CIPN, we found biologically active candidates to relieve CIPN from traditionally used medicinal herb extracts. In the present study, we demonstrated the neuroprotective effects of EFSF on OIPN; EFSF alleviated oxaliplatin-induced neurotoxicity which was observed both in vitro neurotoxicity model using the neural PC12 cells and in vivo OIPN mouse model. In addition, we also found that FSA, a major component of EFSF, could protect the neurotoxicity against oxaliplatin. Taken together, our results suggest that EFSF and FSA are useful for relieving OIPN and could be a possible candidate to develop a new supportive drug for the cancer patients receiving oxaliplatin treatment.

To examine whether EFSF can protect against the chemotherapy-induced neurotoxicity, we first performed a cell viability assay with neural PC12 cells after co-treatment with EFSF and anticancer drugs, including oxaliplatin, cisplatin, docetaxel, paclitaxel, vincristine, and bortezomib. All the anticancer drugs tested induced cytotoxicity in the neural PC12 cells; EFSF treatment effectively attenuated the anticancer drug-induced cytotoxicity of neural PC12 cells. Among them, oxaliplatin induced a potent neurotoxicity, showing a remarkable decrease in the cell viability as well as neuronal differentiation of the neural PC12 cells based on the number of cells bearing neurites and the total length of neurites. EFSF attenuated the oxaliplatin-induced neurotoxicity in a dose-dependent manner; a high dose of EFSF (100 μg/ml) could protect almost completely the neural PC12 cells from the oxaliplatin-induced neurotoxicity. Because there is a correlation between accumulation of oxaliplatin in DRG neurons and neurotoxicity, DRG is a primary target of oxaliplatin-mediated peripheral neurotoxicity [38, 39]. In this study, we examined whether EFVF affects the oxaliplatin-induced neurotoxicity in primary rat DRG cells, and confirmed that EFVF has neuroprotective effects against oxaliplatin-induced neurotoxicity based on the results of neurite outgrowth assay. Our previous study has shown that oxaliplatin dramatically decreased the cell viability and induced apoptosis of primary cultured DRG cells, which results in inhibition of neurite outgrowth [30]. Taken together, these data suggest that EFSF can exert its neuroprotective effect in primary DRG cells as well as neuronal differentiated PC12 cells.

In traditional Korean medicine, both dried fruits of *Forsythia viridissima* and *Forsythia suspensa* have been used as Forsythiae Fructus [1]. It has been known that these two medicinal plants belonged to the genus *Forsythia,* but the constituents of their dried fruits were different; The major components of *Forsythia suspensa* fruits were arctiin, matairesinol, and arctigenin*,* while the major component of *Forsythia viridissima* fruits was forsythoside A [1, 2]. In previous study, we have shown that the aqueous extract of *Forsythia viridissima* fruits (EFVF) has neuroprotective effects against OIPN, and its major components arctiin and arctigenin attenuated the oxaliplatin-induced neurotoxicity [32]. Therefore, this study investigated whether the aqueous extract of *F. suspensa* could exert neuroprotective effects against OIPN.

Several studies have demonstrated that *F. suspensa* has antioxidant and anti-inflammatory properties [17, 40], and its major component, FSA, also possesses anti-inflammatory and anti-oxidant properties [6, 14]. Furthermore, it was also founded that *F. suspensa* or FSA has neuroprotective effects against neurodegenerative diseases such as Parkinson’s disease [17], Alzheimer’s disease [41], learning and memory deficits [42], and cerebral global ischemia [43]. Until recently, however, the protective effects of EFSF and FSA against peripheral neuropathy have not been reported yet. Therefore, as far as we know, our data showed for the first time that EFSF and its major component FSA have a potent protective effect against oxaliplatin-induced neurotoxicity.

Oxaliplatin has a very narrow therapeutic window because of the adverse side effects such as peripheral neuropathy and diarrhea [26, 44]. It has been known that the repeated treatment of oxaliplatin leads to the dysfunction of the sensory neurons, and the degree of OIPN depends on the cumulative dose and the duration of treatment [44–46]. To investigate the effect of EFSF on OIPN, chronic OIPN animal models were established using C57BL/6 mice without a tumor burden for ethical reasons and practical feasibility as previously described [47]. Experimental mice repeatedly received intraperitoneal administration of oxaliplatin until a cumulative dose of oxaliplatin reached to 20–30 mg/kg. These chronic OIPN models were confirmed by measuring the mechanical hypersensitivity using the von Frey test. The mice receiving oxaliplatin did exhibit increased mechanical sensitivity which were assessed by measuring the withdrawal responses of animals to the mechanical stimuli. The behavior test of the neuropathic mice showed that the repeated administration of low dose of oxaliplatin reduced the nociceptive withdrawal threshold and increased the response rate to the mechanical stimuli. Mechanical hypersensitivity induced by oxaliplatin persisted for more than 40 days. In addition a significant loss of IENFs in the digitals which is a pathological marker and indicator of small fiber neuropathy [48, 49] was also in oxaliplatin-treated mice indicating that the present OIPN animal model successfully mimicked the chronic OIPN observed in oxaliplatin-treated cancer patients. In our OIPN mouse model EFSF could dramatically attenuate the oxaliplatin-induced nociceptive hypersensitivity and preserve peripheral nerve fibers in digital skin tissues. Taken together, our data indicate that EFSF has neuroprotective effects against oxaliplatin-induced peripheral neuropathy.

The exact mechanisms underlying the development of OIPN are not yet fully understood; however, it seems that alterations in mitochondria, membrane receptors, ion channels, and inflammatory mediators such as cytokines and chemokines leading to neuroinflammation, oxidative stress, mitochondrial dysfunction, and axonal degeneration are likely involved in the pathogenesis of OIPN [22, 50]. Mannelli and co-workers reported that oxaliplatin-induced neuropathic pain in rat was characterized by a significant oxidative damage throughout the nervous system [51]. They also showed that the natural antioxidants sillbinin and a-tocopherol reduced the oxidative damage in SH-SY5Y and primary rat cortical astrocytes, and protected astrocyte from the oxaliplatin-induced extrinsic apoptosis without affecting its anticancer activity in HT-29 colon cancer cells [52]. In addition, they found that oxaliplatin-induced apoptotic signals have a different preference in astrocytes and HT-29 cancer cells; the intrinsic pathway prevails in normal nervous cells but the extrinsic apoptosis signal in tumor cells [53]. As described above, since *F. suspensa* and its major component, FSA both possess anti-inflammatory and anti-oxidant properties [6, 14], it suggested that EFSF and FSA could affect the oxaliplatin-induced neuroinflammation, oxidative stress, mitochondrial dysfunction, or axonal degeneration. To elucidate the mechanism of the neuroprotective effect of *F. suspensa* and FSA against oxaliplatin-induced neurotoxicity, the effect of ESFS or FSA on changes in apoptotic signaling pathways, mitochondrial function, membrane receptors, and release of cytokines and chemokines should be investigated as a further study.

Because adjuvant drugs may affect the antitumor activity of anticancer drugs, in general, the drug-drug interactions between the cancer drug and supportive drug to be co-administered should be evaluated during the development of cancer supportive drugs. The present study showed that EFSF did not have any negative effect on the antitumor activity profiles of oxaliplatin at least in human colorectal (HCT-116) and lung cancer (A549) cells. Therefore, it can be expected that EFSF may exert a neuroprotective effect against OIPN without affecting the anti-tumor effect of oxaliplatin itself. In order to develop EFSF as a therapeutic agent for OIPN, further studies on the precise molecular mechanisms of EFSF underlying the development of OIPN, and on the herb-drug interaction should be preceded.

## Conclusions

The present study showed that EFSF could alleviate oxaliplatin-induced neurotoxicity in vitro and peripheral neuropathy in vivo suggesting that it could be a starting material to develop a potential functional food ingredient or a supportive drug for managing of cancer patients receiving chemotherapy with oxaliplatin.

## Data Availability

Data are all contained within the paper and materials are available from the senior author.

## Abbreviations

CIPN: Chemotherapy-induced peripheral neuropathy
DAD: Diode array detector
DRG: Dorsal root ganglion
EFSF: Aqueous extract of *F. suspensa* fruits
FSA: Forsythoside A
IC_50_: Half maximal inhibitory concentration
IENF: Intra-epidermal nerve fiber
LOHP: Oxaliplatin
NGF: Nerve growth factor
OIPN: Oxaliplatin-induced peripheral neuropathy
PN: Peripheral neuropathy
STD: Authentic standard chemical
UHPLC: Ultra-high performance liquid chromatography

## References

[1] Korea Food and Drug Administration. South Korean pharmacopoeia; Monografs part II. Se jong. South Korea: Ministry of Health and Welfare; 2015.

[2] Chinese Pharmacopoeia Commission. Pharmacopoeia of the People’s Republic of China. Beijing, China: Chemical Industry Press; 2015.

[3] Dong Zhanglu, Lu Xianyuan, Tong Xueli, Dong Yaqian, Tang Lan, Liu Menghua (2017). Forsythiae Fructus: A Review on its Phytochemistry, Quality Control, Pharmacology and Pharmacokinetics. Molecules.

[4] Wang ZY, Xia Q, Liu X, Liu WX, Huang WZ, Mei X, Luo J, Shon MX, Lin RC, Zou DX (2018). Phytochemistry, pharmacology, quality control and future research of *Forsythia suspensa* (Thunb.) Vahl: a review. J Ethnopharmacol.

[5] Bao JL, Ding RB, Zou LD, Zhang C, Wang K, Liu F, Li P, Chen MW, Wan JB, Su HX (2016). Forsythiae Fructus inhibits B16 melanoma growth involving MAPKs/Nrf2/HO-1 mediated anti-oxidation and anti-inflammation. Am J Chin Med.

[6] Cheng G, Zhao Y, Li H, Wu Y, Li X, Han Q, Dai C, Li Y (2014). Forsythiaside attenuates lipopolysaccharide-induced inflammatory responses in the bursa of Fabricius of chickens by downregulating the NF-kappaB signaling pathway. Exp Ther Med.

[7] Hao Y, Li D, Piao X, Piao X (2010). *Forsythia suspensa* extract alleviates hypersensitivity induced by soybean beta-conglycinin in weaned piglets. J Ethnopharmacol.

[8] Lee Ji Yun, Moon Hee, Kim Chang Jong (2010). Effects of Hydroxy Pentacyclic Triterpene Acids from Forsythia viridissima on Asthmatic Responses to Ovalbumin Challenge in Conscious Guinea Pigs. Biological & Pharmaceutical Bulletin.

[9] Lee JH, Lee JY, Kim TD, Kim CJ. Antiasthmatic action of dibenzylbutyrolactone lignans from fruits of *Forsythia viridissima *on asthmatic responses to ovalbumin challenge in conscious Guinea-pigs. Phytother Res. 2011;25(3):387–95. 10.1002/ptr.3273 20734328

[10] Cho SH, Rhee SJ, Choi SW, Choi Y (2004). Effects of forsythia fruit extracts and lignan on lipid metabolism. Biofactors..

[11] Guo Hui, Liu Ai-Hua, Li Lie, Guo De-An (2007). Simultaneous determination of 12 major constituents in Forsythia suspensa by high performance liquid chromatography—DAD method. Journal of Pharmaceutical and Biomedical Analysis.

[12] Fang Xinsheng, Gu Shubo, Jin Zongyuan, Hao Mingqian, Yin Zhenzhen, Wang Jianhua (2018). Optimization of Ultrasonic-Assisted Simultaneous Extraction of Three Active Compounds from the Fruits of Forsythia suspensa and Comparison with Conventional Extraction Methods. Molecules.

[13] Wang Y, Zhao H, Lin C, Ren J, Zhang S (2016). Forsythiaside a exhibits anti-inflammatory effects in LPS-stimulated BV2 microglia cells through activation of Nrf2/HO-1 signaling pathway. Neurochem Res.

[14] Huang CK, Lin YL, Su H, Ye DQ (2015). Forsythiaside protects against hydrogen peroxide-induced oxidative stress and apoptosis in PC12 cell. Neurochem Res.

[15] Qu H, Zhang Y, Wang Y, Li B, Sun W (2008). Antioxidant and antibacterial activity of two compounds (forsythiaside and forsythin) isolated from *Forsythia suspensa*. J Pharm Pharmacol.

[16] Sung YY, Yoon T, Jang S, Kim HK (2016). *Forsythia suspensa* suppresses house dust mite extract-induced atopic dermatitis in NC/Nga mice. PLoS One.

[17] Zhang Shuai, Shao Si-Yuan, Song Xiu-Yun, Xia Cong-Yuan, Yang Ya-Nan, Zhang Pei-Cheng, Chen Nai-Hong (2016). Protective effects of Forsythia suspense extract with antioxidant and anti-inflammatory properties in a model of rotenone induced neurotoxicity. NeuroToxicology.

[18] Jaggi AS, Singh N (2012). Mechanisms in cancer-chemotherapeutic drugs-induced peripheral neuropathy. Toxicology.

[19] Alcindor T, Beauger N (2011). Oxaliplatin: a review in the era of molecularly targeted therapy. Curr Oncol.

[20] Cavaletti G, Marmiroli P (2015). Chemotherapy-induced peripheral neurotoxicity. Curr Opin Neurol.

[21] Zhang X, Chen WW, Huang WJ (2017). Chemotherapy-induced peripheral neuropathy. Biomed Rep.

[22] Starobova H, Vetter I (2017). Pathophysiology of chemotherapy-induced peripheral neuropathy. Front Mol Neurosci.

[23] Argyriou AA, Cavaletti G, Briani C, Velasco R, Bruna J, Campagnolo M, Alberti P, Bergamo F, Cortinovis D, Cazzaniga M (2013). Clinical pattern and associations of oxaliplatin acute neurotoxicity: a prospective study in 170 patients with colorectal cancer. Cancer.

[24] Saif MW, Reardon J (2005). Management of oxaliplatin-induced peripheral neuropathy. Ther Clin Risk Manag.

[25] Grothey A (2005). Clinical management of oxaliplatin-associated neurotoxicity. Clin Colorectal Cancer.

[26] Cersosimo RJ (2005). Oxaliplatin-associated neuropathy: a review. Ann Pharmacother.

[27] Chu SH, Lee YJ, Lee ES, Geng YM, Wang XS, Cleeland CS (2015). Current use of drugs affecting the central nervous system for chemotherapy-induced peripheral neuropathy in cancer patients: a systematic review. Support Care Cancer.

[28] Olsen Y, The CDC (2016). Guideline on opioid prescribing rising to the challenge. Jama-J Am Med Assoc.

[29] Smith TJ, Saiki CB (2015). Cancer pain management. Mayo Clin Proc.

[30] Majithia N, Loprinzi CL, Smith TJ (2016). New practical approaches to chemotherapy-induced neuropathic pain: prevention, assessment, and treatment. Oncology.

[31] Tofthagen C, Visovsky CM, Hopgood R (2013). Chemotherapy-induced peripheral neuropathy: an algorithm to guide nursing management. Clin J Oncol Nurs.

[32] Yi Jin-Mu, Shin Sarah, Kim No, Bang Ok-Sun (2019). Neuroprotective Effects of an Aqueous Extract of Forsythia viridissima and Its Major Constituents on Oxaliplatin-Induced Peripheral Neuropathy. Molecules.

[33] Cho ES, Yi JM, Park JS, Lee YJ, Lim CJ, Bang OS, Kim NS (2016). Aqueous extract of Lithospermi radix attenuates oxaliplatin-induced neurotoxicity in both in vitro and in vivo models. BMC Complement Altern Med.

[34] Shin MK (1996). Clinical Traditional Herbalogy.

[35] Nair AB, Jacob S (2016). A simple practice guide for dose conversion between animals and human. J Basic Clin Pharm.

[36] Thomas ES, Elizabeth MG, Harvey IP, Giorgio VS, David B (2018). 43 neurotoxicity related to radiotherapy and chemotherapy for nonsmall cell and small cell lung Cancer. IASLC thoracic oncology.

[37] Han Y, Smith MT (2013). Pathobiology of cancer chemotherapy-induced peripheral neuropathy (CIPN). Front Pharmacol.

[38] Ceresa C, Avan A, Giovannetti E, Geldof AA, Avan A, Cavaletti G, Peters GJ (2014). Characterization of and protection from neurotoxicity induced by oxaliplatin, bortezomib and epothilone-B. Anticancer Res.

[39] Ta LE, Espeset L, Podratz J, Windebank AJ (2006). Neurotoxicity of oxaliplatin and cisplatin for dorsal root ganglion neurons correlates with platinum-DNA binding. Neurotoxicology.

[40] Zhao PF, Piao XS, Pan L, Zeng ZK, Li QY, Xu X, Wang HL (2017). *Forsythia suspensa* extract attenuates lipopolysaccharide-induced inflammatory liver injury in rats via promoting antioxidant defense mechanisms. Anim Sci J.

[41] Yan X, Chen T, Zhang L, Du H (2017). Protective effects of Forsythoside a on amyloid beta-induced apoptosis in PC12 cells by downregulating acetylcholinesterase. Eur J Pharmacol.

[42] Wang HM, Wang LW, Liu XM, Li CL, Xu SP, Farooq AD (2013). Neuroprotective effects of forsythiaside on learning and memory deficits in senescence-accelerated mouse prone (SAMP8) mice. Pharmacol Biochem Behav.

[43] Kim JM, Kim S, Kim DH, Lee CH, Park SJ, Jung JW, Ko KH, Cheong JH, Lee SH, Ryu JH (2011). Neuroprotective effect of forsythiaside against transient cerebral global ischemia in gerbil. Eur J Pharmacol.

[44] Krishnan AV, Goldstein D, Friedlander M, Kiernan MC (2005). Oxaliplatin-induced neurotoxicity and the development of neuropathy. Muscle Nerve.

[45] Mihara Y, Egashira N, Sada H, Kawashiri T, Ushio S, Yano T, Ikesue H, Oishi R (2011). Involvement of spinal NR2B-containing NMDA receptors in oxaliplatin-induced mechanical allodynia in rats. Mol Pain.

[46] Pasetto LM, D'Andrea MR, Rossi E, Monfardini S (2006). Oxaliplatin-related neurotoxicity: how and why?. Crit Rev Oncol Hematol.

[47] Hopkins HL, Duggett NA, Flatters SJL (2016). Chemotherapy-induced painful neuropathy. Pain-like behaviours in rodent models and their response to commonly used analgesics. Curr Opin Support Pa.

[48] Koskinen MJ, Kautio AL, Haanpaa ML, Haapasalo HK, Kellokumpu-Lehtinen PL, Saarto T, Hietaharju AJ. Intraepidermal nerve fibre density in cancer patients receiving adjuvant chemotherapy. Anticancer Res. 2011;31(12):4413–6. 22199308

[49] Meyer L, Patte-Mensah C, Taleb O, Mensah-Nyagan AG (2011). Allopregnanolone prevents and suppresses oxaliplatin-evoked painful neuropathy: multi-parametric assessment and direct evidence. Pain.

[50] Zajaczkowska R, Kocot-Kepska M, Leppert W, Wrzosek A, Mika J, Wordliczek J (2019). Mechanisms of chemotherapy-induced peripheral neuropathy. Int J Mol Sci.

[51] Di Cesare ML, Zanardelli M, Failli P, Ghelardini C (2012). Oxaliplatin-induced neuropathy: oxidative stress as pathological mechanism: protective effect of silibinin. J Pain.

[52] Di Cesare ML, Zanardelli M, Failli P, Ghelardini C (2013). Oxaliplatin-induced oxidative stress in nervous system-derived cellular models: could it correlate with in vivo neuropathy?. Free Radical Bio Med.

[53] Zanardelli Matteo, Micheli Laura, Nicolai Raffaella, Failli Paola, Ghelardini Carla, Di Cesare Mannelli Lorenzo (2015). Different Apoptotic Pathways Activated by Oxaliplatin in Primary Astrocytes vs. Colo-Rectal Cancer Cells. International Journal of Molecular Sciences.

